# Comments on “Effects of vitamin D supplementation on the functional outcome in patients with osteoporotic vertebral compression fracture and vitamin D deficiency”

**DOI:** 10.1186/s13018-023-03532-y

**Published:** 2023-02-09

**Authors:** Chong Li, Ke Lu

**Affiliations:** Department of Orthopedics, Affiliated Kunshan Hospital of Jiangsu University, No. 91 West of Qianjin Road, Suzhou, 215300 Jiangsu China

Dear Editor,

Takahiro Niikura et al. [1] investigated the effects of vitamin D supplementation on functional outcomes in patients with osteoporotic vertebral compression fracture (OVCF) and vitamin D deficiency, which piqued our interest. While we appreciate the efforts made by authors in completing this study, we believe some issues should be addressed to strengthen this article.

First, this study examined 130 patients with OVCF who visited their clinic between January 2019 and January 2020 based on the selection criteria. However, the authors did not go into detail about how the OVCFs were handled. Were the patients conservatively or surgically treated? What specific surgical techniques were used in this study (e.g., percutaneous kyphoplasty [PKP])? The omission of such critical intervention information can render the final results unreliable.

Second, the authors claimed that 41 out of 83 patients in the deficient group and 24 out of 47 patients in the insufficient group were in the non-supplementation group that did not receive vitamin D supplementation. However, the specific method of grouping was not clarified. And the use of a non-randomized method of grouping may lead to bias.

Finally, this study used vitamin D supplementation as an intervention factor. As a result, Table 2 shows epidemiological findings divided by vitamin D supplementation, and baseline vitamin D levels for both groups need to be compared.

While the authors of this study presented promising data on 12-month follow-up outcomes of these 130 patients, we believe that further clarification of these issues would significantly improve the article as a whole.
